# Environmental Screening of *Fonsecaea* Agents of Chromoblastomycosis Using Rolling Circle Amplification

**DOI:** 10.3390/jof6040290

**Published:** 2020-11-17

**Authors:** Morgana Ferreira Voidaleski, Renata Rodrigues Gomes, Conceição de Maria Pedrozo e Silva de Azevedo, Bruna Jacomel Favoreto de Souza Lima, Flávia de Fátima Costa, Amanda Bombassaro, Gheniffer Fornari, Isabelle Cristina Lopes da Silva, Lucas Vicente Andrade, Bruno Paulo Rodrigues Lustosa, Mohammad J. Najafzadeh, G. Sybren de Hoog, Vânia Aparecida Vicente

**Affiliations:** 1 Postgraduate Program in Microbiology, Parasitology and Pathology, Biological Sciences, Department of Basic Pathology, Federal University of Parana, Curitiba 81531-980, Brazil; morganavoidaleski@gmail.com (M.F.V.); rrgrenata@gmail.com (R.R.G.); jacomel.bruna@gmail.com (B.J.F.d.S.L.); amandabssaro@gmail.com (A.B.); isabelle.lopes18@gmail.com (I.C.L.d.S.); 2 Department of Medicine, Federal University of Maranhão, Vila Bacanga, Maranhão 65080-805, Brazil; conceicaopedrozo@gmail.com; 3 Bioprocess Engineering and Biotechnology, Federal University of Paraná, Curitiba 82590-300, Brazil; flaviafc88@gmail.com (F.d.F.C.); brunopaulorl@gmail.com (B.P.R.L.); 4 Real Field College, Biomedicine Course, Guarapuava 85015-240, Brazil; gheniffer.fornari@gmail.com; 5 União das Faculdades dos Grandes Lagos, Medical College, Clinic Medical, São José do Rio Preto 15030-070, SP, Brazil; vicente_lva@hotmail.com; 6 Department of Parasitology and Mycology, School of Medicine, Mashhad University of Medical Sciences, Mashhad 9177948564, Iran; najafzadehmj@mums.ac.ir; 7 Center of Expertise in Mycology, Radboud University Medical Center/Canisius Wilhelmina Hospital, 6525 GA Nijmegen, The Netherlands

**Keywords:** Black yeast, padlock probe, *Fonsecaea pedrosoi*, *Fonsecaea monophora*

## Abstract

Chromoblastomycosis is a chronic, cutaneous or subcutaneous mycosis characterized by the presence of muriform cells in host tissue. Implantation disease is caused by melanized fungi related to black yeasts, which, in humid tropical climates, are mainly members of the genus *Fonsecaea*. In endemic areas of Brazil, *F. pedrosoi* and *F. monophora* are the prevalent species. The current hypothesis of infection is traumatic introduction via plant materials, especially by plant thorns. However, isolation studies have demonstrated a low frequency of the agents in environmental substrates. The present study aimed to detect *F. pedrosoi* and *F. monophora* in shells of babassu coconuts, soil, plant debris, and thorns from endemic areas of chromoblastomycosis in Maranhão state, northern Brazil, using Rolling Circle Amplification (RCA) with padlock probes as a new environmental screening tool for agents of chromoblastomycosis. In addition to molecular screening, the environmental samples were analyzed by fungal isolation using mineral oil flotation. The limit of detection of the RCA method was 2.88 × 10^7^ copies of DNA per sample for the used padlock probes, indicating that this represents an efficient and sensitive molecular tool for the environmental screening of *Fonsecaea* agents. In contrast, with isolation from the same samples using several selective methods, no agents of chromoblastomycosis were recovered.

## 1. Introduction

Melanized fungi in the family Herpotrichiellaceae (order Chaetothyriales) are involved in persistent (sub) cutaneous human infections [1]. These fungi have a complex ecological preference and life cycle, which are still poorly understood. They may be found in adverse and extreme conditions, such as on rocks, in arid and hot climates, and in toxic habitats, and also occur as opportunistic pathogens [2,3]. Virulence factors such as the presence of melanin and carotene, thick cell walls, differentiation in muriform cells, yeast phases, osmotolerance, adhesion, hydrophobicity, the assimilation of aromatic hydrocarbons, and the production of siderophores are shared by many members of the family and therefore contribute to the opportunistic nature of these infectious agents [4,5].

Chromoblastomycosis (CBM) is an uncommon and chronic, cutaneous and subcutaneous disease that mostly occurs in immunocompetent patients, resulting in nodular deformations and the presence of muriform cells in tissue, leading to a granulomatous immune response [6]. CBM is considered an occupational disease, especially among agricultural workers, and is caused by the transcutaneous implantation of plant material in tropical and subtropical climate zones around the world [6]. CBM lesions are usually recalcitrant and extremely difficult to eradicate. The disease mainly affects the poorest populations that often live in remote rural areas and have little political support to achieve priority in public health systems. Therefore, the disease has received the status of a neglected tropical disease by the World Health Organization (WHO) [7]. Species that have been described to cause cutaneous infection are classified as *Exophiala*, *Cyphellophora*, *Phialophora*, and *Rhinocladiella*, but the main agents of CBM belong to *Cladophialophora* and *Fonsecaea*. Members of the latter genera have a different epidemiology: *Fonsecaea* species are primarily found in humid areas, whereas *Cladophialophora carrionii* is prevalent in semiarid climates [6,8,9,10]. In Brazil, the Amazonian region is considered an endemic area, with the Maranhão state being hyperendemic, exhibiting the largest number of records of the disease [9,11,12]. According to the literature, *Fonsecaea pedrosoi* is the main etiological agent in these areas, followed by *Fonsecaea monophora*, which is also known from brain infections [12].

Epidemiological data on mycosis suggest an environmental origin [7,11]. The infection is frequently reported following the occurrence of skin trauma, mainly by plant thorns or wood fragments [13]. Knowledge of the environmental occurrence of pathogenic agents is important in understanding infection pathways, but methods of isolation remain an obstacle [14]. Studies have shown that species recovered from living plants or plant debris often belong to non-pathogenic relatives of the agents of chromoblastomycosis [11,15,16]. A comparative genomic analysis of *Fonsecaea* species demonstrated that environmental and opportunistic species share gene domains associated with the invasion of plant tissue, supporting the hypothesis of traumatic inoculation from plant material [17].

Molecular markers provide a reliable tool for characterizing the habitat of clinical species, presenting a high specificity, reproducibility, and sensitivity. Through the extraction of total DNA from environmental samples, it becomes possible to evaluate the fungal diversity in the sample, enhancing phylogenetic interference, the taxonomic delimitation of species, and identification [18,19,20,21]. In order to elucidate epidemiological aspects, efficient methods are required to recover and characterize pathogenic agents [6,18,22]. Padlock probes are oligonucleotides containing around 100 bp that recognize single point polymorphisms (SNPs) in target DNA in large populations of over 500 individuals [22,23,24]. Ligation products of the padlock probe can be amplified by isothermal amplification in Rolling Circle Amplification (RCA), which represents a sensitive and specific molecular method for the detection of *Fonsecaea* species [25].

The present study proposes the use of RCA padlock probes, previously described in the literature [25], as a new strategy for the environmental screening of *F. pedrosoi* and *F. monophora*, which are the main agents of chromoblastomycosis in endemic areas of Maranhão state, Brazil, in order to elucidate the routes of infection and ecological niches of species related to the disease.

## 2. Materials and Methods

### 2.1. Study Area and Samples

**Figure 1:**
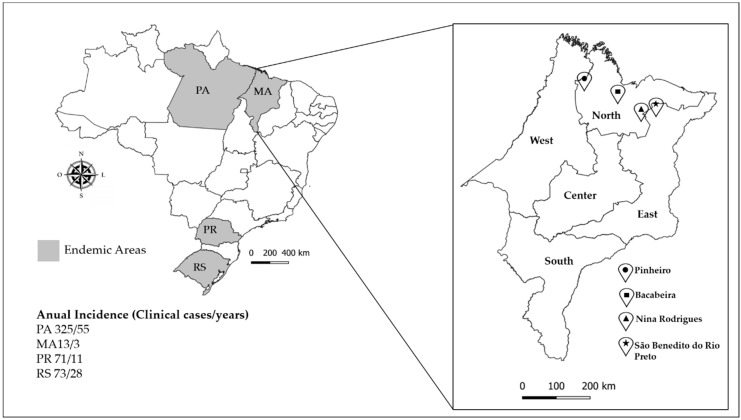
Study area of Maranhão state. The annual incidence of chromoblastomycosis in Maranhão state and endemic areas is shown in gray on the map [6]. The area of study was located in the North and East of Maranhão state. Bacabeira, latitude: 02° 58′ 15″ S; longitude: 44° 18′ 56″ W; altitude: 44 m; area: 650 km^2^. São Benedito do Rio Preto, latitude: 3° 19′ 59″ S; longitude: 43° 31′ 40″ W; altitude: 22 m; area: 931.48 km^2^. Pinheiro, latitude: 02° 31′ 17″ S; longitude: 45° 04′ 57″ W; altitude: 15 m; area: 1559 km^2^. Nina Rodrigues, latitude: 3° 27′ 53″ S; longitude: 43° 54′ 19″ W; altitude: 33 m; area: 572.5 km^2^.

A total of 87 environmental samples were collected randomly in the living environment of symptomatic patients, i.e., five samples of each environmental source (soil, decomposing plant material, and living plants), of each one of the four regions in the north of Maranhão state (Figure 1). Plant materials of *Solanum paniculatum* (Jurubeba tree), *Astrocaryum vulgare* (Tucum tree), *Platonia insignis* (Bacuri tree), *Scoparia dulcis* (Vassourinha tree), *Murraya paniculata* (Murta tree), and *Urtica* spp. were divided according to leaves, stems, and thorns (when present). About 30 g of each sample was collected separately in sterilized paper bags [12,26]. In addition, 20 DNA samples of the babassu coconut shell, which is a known source of melanized fungi [2] and which has been suggested as a possible risk factor for CBM [26,27], were included.

### 2.2. Padlock Probes

#### 2.2.1. DNA Extraction of Environmental Samples

About 250 mg of each environmental sample was transferred to a 2 mL microtube containing 300 µL cetyltrimethylammonium bromide (CTAB) and about 80 mg of a silica mixture. Cells were grinded manually with a sterile pestle for approximately 5 min. Subsequently, 700 µL CTAB buffer was added. The mixture was vortexed for 5 min and incubated for 60 min at 65 °C. Then, 600 μL 24:1 chloroform: isoamylalcohol was added, mixed carefully, and centrifuged for 10 min at 12,000× *g* force. The supernatant was transferred to a new tube and 800 µL ice-cold 100% isopropyl alcohol was added. DNA was allowed to precipitate for 45 min at −20 °C and then centrifuged again for 15 min at 12,000× *g*. The pellet was washed twice with 500 µL cold 70% ethanol and once with 500 µL of cold 100% ethanol. After drying at room temperature, samples were resuspended in 100 µL of ultrapure water. The purity and integrity of the DNA were evaluated by spectrophotometry (NanoDrop^®^, Thermo Scientific, Waltham, MA, EUA) and on agarose gel 1% [16,28]. Total DNA extraction from the soil samples was performed using the EZNA Soil DNA kit (Omega Bio-Tek, Norcross, GA, USA).

#### 2.2.2. DNA Amplification

Reaction mixtures had a total volume of 12.5 μL, comprising 1× PCR buffer, 2.0 mM MgCl_2_, 25 μM deoxynucleoside triphosphates (dNTPs), 0.5 μM of each forward and reverse primers ITS 1 and ITS 4 [29], 1 U of Taq DNA polymerase (Ludwing Biotec, Bela Vista, Brazil), and 20 ng of genomic DNA. Amplification was performed in an ABI Prism 2720 thermocycler (Applied Biosystems, Foster City, CA, USA), as follows: 95 °C for 4 min, followed by 35 cycles consisting of 95 °C for 45 s, 52 °C for 30 s, and 72 °C for 2 min, and a delay at 72 °C for 7 min. For some samples, annealing temperatures were changed from 50 to 55 °C.

#### 2.2.3. Ligation of Padlock Probes

The padlock probes FOP (*F. pedrosoi*) and FOM (*F. monophora*) used in this study were previously designed by Najafzadeh et al. in 2011 [25]. One microliter of ITS amplicon was mixed with 2 U pfu DNA ligase (Agilent Technologies, Santa Clara, CA, USA) and 0.1 µmol l^−1^ padlock probe in 20 mmol l^−1^ Tris-HCl (pH 7.5), 20 mmol l^−1^ KCl, 10 mmol l^−1^ MgCl2, 0.1% Igepal, 0.01 mmol l^−1^ rATP, and 1 mmol l^−1^ DTT, with a total reaction volume of 10 µL. Padlock probe ligation was conducted with one cycle of denaturation for 5 min at 94 °C, followed by five cycles of 94 °C for 30 s and 4 min ligation at 50 °C. An exonucleolysis step was not required.

#### 2.2.4. Rolling Circle Amplification Reaction

One microliter of ligation product was used as a template for RCA. The total volume was 12 µL^−l^, containing 8 U Bst DNA polymerase (New England Biolabs, Ipswich, MA, EUA), 400 µmol L^−l^ deoxynucleoside triphosphate mix, 25 μmol deoxynucleoside triphosphates (dNTPs), and 10 pmol of each RCA primer (RCA1 5′-ATGGGCACCGAAGAAGCA-3′ and RCA2 5′-CGCGCAGACACGATA-3′) in distilled water. Probe signals were amplified by incubation at 65 °C for 60 min, and the accumulation of double stranded DNA products was visualized on a 2% agarose gel to verify the specificity of probe template binding. Positive reactions exhibited a ladder-like pattern, whereas negative reactions displayed a clean background.

#### 2.2.5. Specificity and Detection Limit of RCA Padlock Probes In Vitro and In Vivo

The specificity of the padlock probes was tested for the detection of *F. pedrosoi* and *F. monophora* in environmental samples (Table 1). The DNA of *F. pedrosoi* (CBS 271.37) and *F. monophora* (CBS 269.37) was used as a positive control, and the DNA of *Fonsecaea erecta* (CBS 125760) was employed as a negative control. The effectivity of RCA padlock probes was demonstrated using artificial DNA sample mixtures containing plant debris or soil (20 ng/µL) with fungal DNA (0.5 ng/µL). In vivo, suspensions of a concentration of 10^5^ cells/mL of the positive and negative controls mentioned above were inoculated by direct injection into the stem of the *Bactris gasipaes* (Peach palm). The plants were cultivated in a vase; after 60 days, stem fractions of the plant were collected for total DNA extraction and analysis by RCA.

The sensitivity of padlock probes was tested using different dilutions of the internal transcribed region (ITS) amplicons. ITS concentrations of *F. pedrosoi* and *F. monophora* were determined with a NanoDrop spectrophotometer (Thermo Scientific, Waltham, MA, EUA) and diluted to a final concentration of 2 ng/μL. Copy numbers were calculated with an online tool based on Avogadro’s number (http://cels.uri.edu/gsc/cndna.html). For calculation, an amplicon length of 645 bp was assumed for *F. pedrosoi* and 644 bp for *F. monophora*. We evaluated the sensitivity of the padlock probes to ensure reliable amplification at low levels of target DNA. We performed 10-fold serial dilutions of ITS DNA, starting with 2.88 × 10^9^ copies per tube and ending with 28.8 copies per tube.

### 2.3. Isolation and Molecular Identification

#### 2.3.1. Isolation

Selective isolation was performed [16,30] for positive samples in the RCA padlock probe essay. Approximately 20 g from each sample was processed for fungal isolation. Samples were incubated at room temperature for 30 min in 100 mL of a sterilized saline solution containing 200 U penicillin, 200 μg/L streptomycin, 200 μg/L chloramphenicol, and 500 μg/L cycloheximide. Twenty milliliters of sterilized mineral oil was added to the solution, vigorously shaken for 5 min, and left to settle for 40 min. The oil–water interphase was then collected, inoculated onto Mycosel Agar (Difco), and incubated for 4 weeks at 28 °C, with five replicates per sample. One hundred and seven samples were collected (20 of shell of the babassu coconut, 20 of soil, 20 of plant debris, 20 of leaves, 20 of stems, and 7 of thorns), with a total of 435 replicates.

#### 2.3.2. Molecular Identification

The DNA extraction of fungi isolates was performed [11]. About 1 cm^2^ mycelium of 20 to 30-d-old cultures was transferred to a 2 mL Eppendorf tube containing 300 µL CTAB buffer (CTAB 2% (*w/v*), NaCl 1.4 M, Tris-HCl 100 mM, pH 8.0; EDTA 20 mM, b-mercaptoethanol 0.2% (*v/v*)) and about 80 mg of a silica mixture (silica gel H, Merck, Darmstadt, Germany / Celite 545, Biotec, São Paulo, SP, Brazil, 2:1, *w/w*). Cells were grinded manually with a sterile pestle for approximately 5 min. Subsequently, 200 µL CTAB buffer was added; the mixture was vortexed and incubated for 10 min at 65 °C. After the addition of 500 µL 24:1 chloroform:isoamylalcohol, the solution was mixed and centrifuged for 5 min at 20,500× *g* and the supernatant was transferred to a new tube with two volumes of ice-cold 96% ethanol. DNA was allowed to precipitate for 30 min at −20 °C and then centrifuged again for 5 min at 20,500× *g*. Subsequently, the pellet was washed with cold 70% ethanol. After drying at room temperature, it was resuspended in 100 µL in ultrapure water.

Amplification of the internal transcribed region (ITS) was performed as previously described [12]. Amplicons were subjected to direct sequencing, as follows: 95 °C for 1 min, followed by 30 cycles consisting of 95 °C for 10 s, 50 °C for 5 s, and 60 °C. The sequences obtained were aligned using Mega 7 software and compared to the Isham Barcoding Database (http://its.mycologylab.org/) and GenBank Blast (NCBI https://blast.ncbi.nlm.nih.gov/Blast.cgi).

## 3. Results

**Figure 2:**
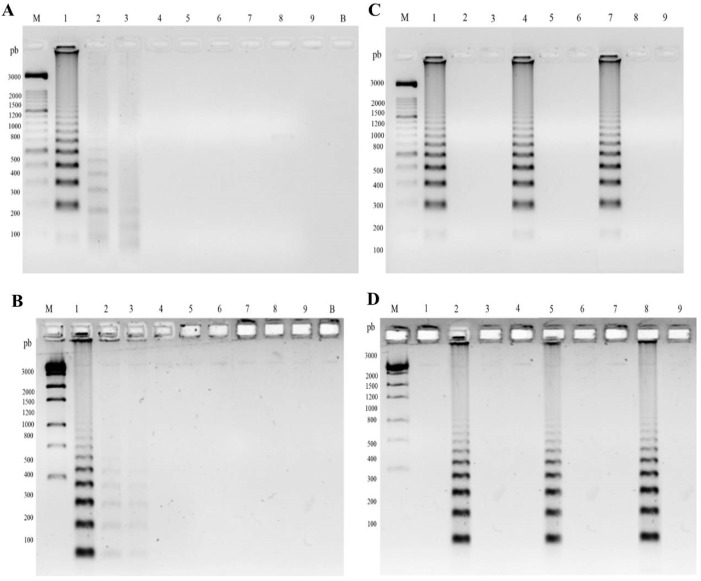
Specificity and limit of detection of padlock probes FOP and FOM by Rolling Circle Amplification (RCA). (**A**) Sensibility of padlock probe FOP amplified by RCA in internal transcribed region (ITS) amplicons of *F. pedrosoi* (CBS 271.37). (**B**) Sensibility of padlock probe FOM amplified by RCA in ITS amplicons of *F. monophora* (CBS 269.37). M, molecular marker 1 Kb; B, blank; 1 to 9, 2.88 × 10^9^, 2.88 × 10^8^, 2.88 × 10^7^, 2.88 × 10^6^, 2.88 × 10^5^, 2.88 × 10^4^, 2.88 × 10^3^, and 2.88 × 10^2^, 2.88 × 10^1^ copies per tube, respectively. (**C,D**) Specificity of padlock probe FOP and FOM, respectively, amplified by RCA in plant and soil samples. In vitro specificity analysis: 1–3, total DNA of plant *Mimosa pudica*; 4–6, total DNA of soil, *F. pedrosoi*, *F. monophora*, and *Fonsecaea erecta*, respectively; 7–9, in vivo specificity analysis of *Bactris gasipaes* in a vessel after injury with *F. pedrosoi*, *F. monophora*, and *F. erecta*, respectively.

The sensitivity of the RCA padlock probes FOP and FOM was determined using serial dilutions of the *F. pedrosoi* (CBS 271.37) and *F. monophora* (CBS 269.37) DNA as templates, respectively. The oligonucleotides ITS1 and ITS4 showed a product of approximately 644 bp. *Fonsecaea pedrosoi* and *F. monophora* were detected at 2.88 × 10^7^ copies of purified DNA (Figure 2A,B). A decrease in the signal intensity was observed at lower concentrations for both probes.

The in vitro specificity analysis demonstrated that the FOP and FOM probe species remained without cross-reaction with closely related species *F. nubica*, *F. erecta*, and *F. pugnacius*, as well as reference DNAs of *Candida albicans*, *Penicillium citrinum*, and *Aspergillus nidulans* (Table 1). The presence of plant material, soil, and other components of total DNA did not interfere with the specificity of the probes and RCA amplification, as demonstrated by in vitro and in vivo analyses (Figure 2C,D). The used RCA padlock probes (FOP and FOM) [25] were shown to be a specific tool for the environmental detection of *F. pedrosoi* and *F. monophora*, with no cross-reaction being observed. In vitro and in vivo assays confirmed the specificity and applicability of the method for environmental sample screening (Figure 2C,D).

**Figure 3:**
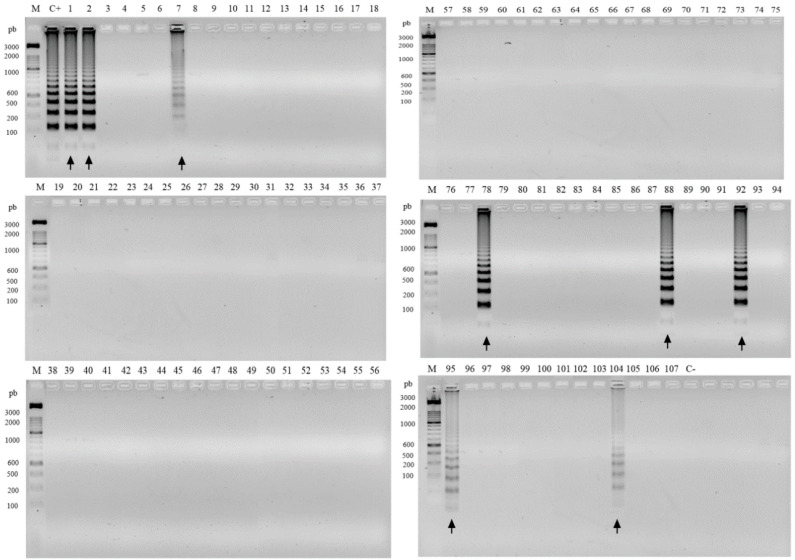
Positive environmental samples for FOP by RCA screening. Molecular marker 1 kb. C+, positive control *F. pedrosoi* (CBS 271.37); C-, negative control *F. erecta* (CBS125760). Bacabeira: 1–5, decomposing plant material; 6–10, leaf; 11–15, thorns; 16–20, steam; 68–72, soil. São Benedito do Rio Preto: 21–25, decomposing plant material; 26–30, leaf; 31–32, thorn; 33–37, steam; 73–77, soil. Pinheiro: 58–62, decomposing plant material; 63–67, leaf; 48–52, steam; 78–82, soil. Nina Rodrigues: 53–57, decomposing plant material; 43–47, leaf; 38–42, steam; 83–87, soil; 88–107, shell of babassu coconut. Positive samples are indicated by arrows.

**Figure 4:**
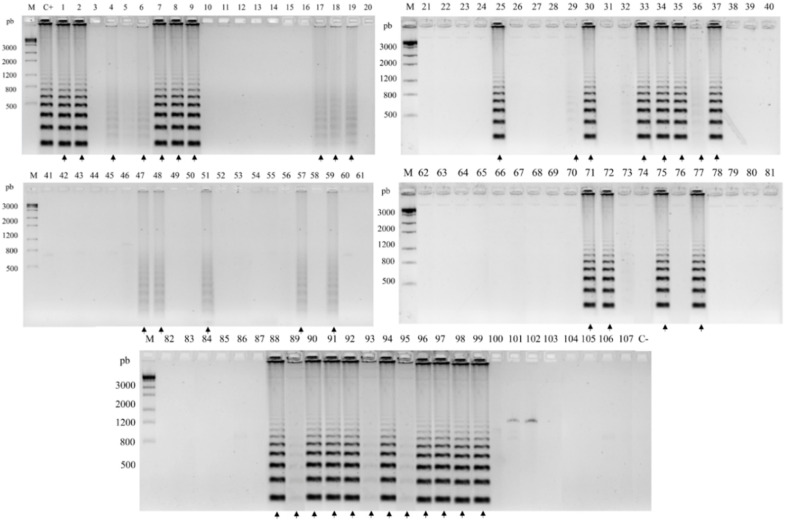
Positive environmental samples for FOM by RCA screening. Molecular marker 1 kb. C+, positive control *F. monophora* (CBS 269.37); C-, negative control *F. erecta* (CBS125760). Bacabeira: 1–5, decomposing plant material; 6–10, leaf; 11–15, thorns; 16–20, steam; 68–72, soil. São Benedito do Rio Preto: 21–25, decomposing plant material; 26–30, leaf; 31–32, thorn; 33–37, steam; 73–77, soil. Pinheiro: 58–62, decomposing plant material; 63–67, leaf; 48–52, steam; 78–82, soil. Nina Rodrigues: 53–57, decomposing plant material; 43–47, leaf; 38–42, steam; 83–87, soil; 88–107, shell of babassu coconut. Positive samples are indicated by arrows.

A total of 107 environmental samples were analyzed by RCA padlock probes (Table S1). Eight (7.48%) of the samples were positive for *F. pedrosoi*, including four samples from shells of babassu coconuts, two from plant debris, one from *Solanum paniculatum* (Jurubeba tree), and one from soil (Figure 3). The FOM padlock probe amplified forty-two (39.25%) of the samples with twelve samples of babassu coconut shell; seven samples of debris plant; twenty samples of plant material of *Murraya paniculata* (Murta tree), *Astrocaryum vulgare* (Tucum tree), *Scoparia dulcis* (Vassourinha tree), and *Platonia insignis* (Bacuri tree); and four samples of soil (Figure 4).

Positive samples for RCA padlock probes were submitted to isolation by oil-mineral flotation. Sequencing of the acquired isolates showed that they belonged to species other than *F. pedrosoi* and *F. monophora*. Judging from the ITS sequencing results, the isolates were affiliated to the order Chaetothyriales in families Trichomeriaceae, Herpotrichiellaceae, and Cyphellophoraceae, and order Capnodiales, families Mycosphaerellaceae and Cladosporiaceae (Table 2).

## 4. Discussion

The epidemiology of chromoblastomycosis suggests that the etiological agents of the disease are present in the environment and the infection is accidental. However, why only a small selection of the apparently saprobic fungi have repeatedly been found in humans, while they theoretically all have a comparative chance of being inoculated, has remained unexplained. Isolates from cactus thorns obtained near a house of a CBM patient had a similar morphology to the common CBM agent *Cladophialophora carrionii*, but by molecular methods, they were identified as *Cladophialophora yegresii*, which has never been found in human hosts [15]. Isolates of both species were demonstrated to be able to form muriform cells when inoculated into cactus plants. Sibling species of CBM agents in *Fonsecaea*, i.e., *F. erecta* and *F. minima*, were commonly associated with plants, but did not have any relationship with chromoblastomycosis [11]. In order to investigate the environmental sources of *F. pedrosoi* and *F. monophora*, substrates reported as possible sources of CBM infection in epidemic areas in Brazil [13,26] were collected, including living plants (leaf, stem, and thorns) and babassu coconut shells.

The RCA probe was previously applied to other causal agents of fungal infection, such as *Sporothrix* spp. [31], *Fusarium graminearum* [32], and *Histoplasma capsulatum* [33]. The limit of detection of the method was 2.88 × 10^7^ copies of DNA for both *F. pedrosoi* and *F. monophora*. A decrease in intensity of the signal amplification was observed when low concentrations of DNA were available, as also reported for *Histoplasma* spp. [33], although this was not observed in *Sporothrix* spp. [31]. The probes for *Fonsecaea* spp. seem to have a higher detection limit, which can be useful for environmental screening.

The isolation of *Fonsecaea* spp. from environmental substrates by oil flotation was reported in several studies [4,11,13,16,26,34,35]. These studies demonstrate that, despite sophisticated selective efforts, the recovery of pathogenic environmental strains is limited. Environmental inoculation is the most parsimonious hypothesis for the onset of chromoblastomycosis, since some patients have presented fragments of plant material in tissue [6,36,37]. Our study shows that the agents of CBM are commonly present in the environment, as are the strictly saprobes species, with some plant material detection unclear. Even though few pathogenic strains are recovered from live plants by conventional isolation methods, these agents of chromoblastomycosis are able to survive in plant tissue after inoculation [38]. The method applied in the present study allowed the detection of DNA of chromoblastomycosis agents in living plants. The presence of *F. pedrosoi* was detected in the leaves of *S. paniculatum* (Jurubeba), in decomposing material, soil, and shells of babassu coconut (*Orbygnia phalerata*) (Figure 3). *Fonsecaea monophora* tests were positive in stems of *M. paniculata* (Murta tree), leaves and stems of *A. vulgare* (Tucum tree), leaves and stems of *S. dulcis* (Vassourinha tree), and leaves of *P. insignis* (Bacuri tree), decomposing material, soil, and shell of babassu coconut (*O. phalerata*) (Figure 4). The isolation by mineral oil flotation was performed in positive samples for RCA, but the chromoblastomycosis agents *F. pedrosoi* and *F. monophora* were not isolated. The babassu coconut has been suggested as a source of agents in Maranhão state of the Brazilian Amazon rainforest [26,27]. Nascimento et al. (2017) [2] isolated numerous chaetothyrialean fungi from this source, but no CBM agents. Our study showed the presence of CBM agents in the DNA from the same study.

The environmental screening of *Fonsecaea* spp. by RCA padlock probes, when compared with selective isolation, demonstrated that CBM agents must be present in environmental samples, even though they were not previously detected [4,11,16,34,35,39,40]. RCA was positive for *F. pedrosoi* and *F. monophora* in most of the substrates analyzed, showing that living plants provide a habitat for the agents, such as *S. paniculatum*, *M. paniculata*, *A. vulgare*, *S. dulcis*, and *P. insignis* (Table S1). This may be explained by the high sensitivity of the probe to detect small concentrations of fungal DNA, even when among competing saprobes. Alternatively, and perhaps more likely, is an explanation by a larger preference for the CBM agents to grow in habitats with similarity to animal tissue; such factors have not yet been revealed. Our results demonstrate that the hypothesis of a route of infection via plant material could be considered, as suggested in clinical reports.

Similar results have been published for environmental pathogens (i.e., pathogens with a double, environment/host alternating life cycle), such as *Histoplasma* and *Paracoccidioides*. Furthermore, these fungi were difficult to isolate from environmental samples, despite positivity with molecular methods [41,42]. The RCA padlock approach applied in this study represents an important method of pathogen detection in environmental samples, contributing to understanding routes of infection. The difference in habitat preference between strict saprobes and opportunistic saprobes remains enigmatic.

## 5. Conclusions

In conclusion, the data obtained in this study showed that the RCA padlock probe represents an efficient, sensitive, and reproducible molecular tool for the environmental screening of opportunistic fungi related to chromoblastomycosis in natural substrates, such as babassu coconut, plants, soil, and decomposing plant material. The use of the padlock probe contributes to new insights into the environmental occurrence and infection route of these agents.

## Figures and Tables

**Table 1:** In vitro specificity analyzes of the FOP and FOM probe species.

DNA Test	Species	Collection Number	Source/Geography	Padlock Probe	
				FOP	FOM
Fungal DNA	*F. pedrosoi*	CBS271.37 ^T^	Chromoblastomycosis, South America	(+)	(−)
	*F. monophora*	CBS269.37 ^T^	Chromoblastomycosis, South America	(−)	(+)
	*F. erecta*	CBS125763 ^T^	Spine of Japecanga plant, Brazil, Bacabeira	(−)	(−)
	*F. nubica*	CBS125.198 ^T^	Chromoblastomycosis, Cameroon	(−)	(−)
	*F. pugnacious*	CMRP1343 ^T^	Chromoblastomycosis, South America	(−)	(−)
	*C. albicans*	CMRP816	Human, Brazil	(−)	(−)
	*P. citrinum*	CMRP1538	Metal, Tucuruí, Brazil	(−)	(−)
	*A. nidulans*	CMRP2338	-	(−)	(−)
Environmental test DNA samples	*M. pudica*		Plant in vitro	(−)	(−)
	*M. pudica* with *F. pedrosoi*	CBS271.37 ^T^	Plant in vitro (20 ng/µL) with 0.5 ng/µL of *F. pedrosoi*	(+)	(−)
	*M. pudica* with *F. monophora*	CBS269.37 ^T^	Plant in vitro (20 ng/µL) with 0.5 ng/µL of *F. monophora*	(−)	(+)
	*M. pudica* with *F. erecta*	CBS125763 ^T^	Plant in vitro (20 ng/µL) with 0.5 ng/µL of *F. erecta*	(−)	(−)
	*B. gasipaes* with *F. pedrosoi*	CBS271.37 ^T^	Plant inoculated with 10^5^ spores of *F. pedrosoi*	(+)	(−)
	*B. gasipaes* with *F. monophora*	CBS269.37 ^T^	Plant inoculated with 10^5^ spores of *F. monophora*	(−)	(+)
	*B. gasipaes* with *F. erecta*	CBS125763 ^T^	Plant inoculated with 10^5^ spores of *F. erecta*	(−)	(−)

^T^, type strain; FOP, *Fonsecaea pedrosoi* padlock probe; FOM, *Fonsecaea monophora* padlock probe; (−), negative sample; (+), positive sample.

**Table 2:** Fungi isolates from positive samples by RCA padlock probes (FOP and FOM).

Positive Sample	Substrate	Padlock Probe Positive	N. Isolates	CMRP	Molecular ID.	GenBank Accession
1	Decomposing Material	FOP; FOM	4	CMRP2566	*Melanoctona tectonae*	MT075634
				CMRP2821	*Melanoctona tectonae*	MT075635
				CMRP2840	*Cladosporium* sp.	MT075636
				CMRP2863	*Cyphellophora* sp.	MT075637
2	Decomposing Material	FOP; FOM	2	CMRP2617	*Strelitziana* sp.	MT080291
				CMRP2859	*Cyphellophora ambigua*	MT075638
4	Decomposing Material	FOM	5	CMRP2619	*Cladosporium* sp.	MT075639
				CMRP2594	*Cladosporium* sp.	MT075640
				CMRP2598	Mycosphaerellaceae	MT080292
				CMRP2826	*Exophiala alcalophila*	MT075641
				CMRP2822	*Exophiala spinifera*	MT075642
6	Leaf, *A. vulgare*	FOM	2	CMRP2601	*Hyalocladosporiella cannae*	MT075643
				CMRP2850	*Exophiala spinifera*	MT075644
7	Leaf, *S. paniculatum*	FOP; FOM	14	CMRP2560	*Hyalocladosporiella cannae*	MT075645
				CMRP2848	*Hyalocladosporiella cannae*	MT075646
				CMRP2564	*Hyalocladosporiella cannae*	MT075647
				CMRP2567	*Hyalocladosporiella cannae*	MT075648
				CMRP2557	*Hyalocladosporiella cannae*	MT075649
				CMRP2589	*Nigrograna obliqua*	MT075650
				CMRP2591	*Hyalocladosporiella cannae*	MT075651
				CMRP2609	*Hyalocladosporiella cannae*	MT075652
				CMRP2615	*Hyalocladosporiella cannae*	MT075653
				CMRP2851	*Hyalocladosporiella cannae*	MT075654
				CMRP2852	*Hyalocladosporiella cannae*	MT075655
				CMRP3098	*Hyalocladosporiella cannae*	MT075656
				CMRP3094	*Hyalocladosporiella cannae*	MT075657
				CMRP2867	*Hyalocladosporiella cannae*	MT075658
8	Leaf, *S. dulcis*	FOM	1	CMRP2868	*Hyalocladosporiella cannae*	MT075659
17	Stalk, *S. paniculatum*	FOM	11	CMRP2562	Chaetothyriales	MT080293
				CMRP2569	*Cyphellophora* sp.	MT075660
				CMRP2620	*Cladosporium* sp.	MT075661
				CMRP2568	*Hyalocladosporiella cannae*	MT075662
				CMRP2586	*Hyalocladosporiella cannae*	MT075663
				CMRP2622	Mycosphaerellaceae	MT080294
				CMRP2614	*Hyalocladosporiella cannae*	MT075664
				CMRP2828	*Strelitziana* sp.	MT080295
				CMRP2837	*Cyphellophora oxyspora*	MT075665
				CMRP2839	*Teratosphaeria* sp.	MT080296
				CMRP3086	Chaetothyriales	MT080297
35	Stalk, *A. vulgare*	FOM	1	CMRP2624	*Strelitziana* sp.	MT080298
47	Leaf, *S. dulcis*	FOM	1	CMRP3082	Mycosphaerellaceae	MT080299
51	Stalk, *S. dulcis*	FOM	6	CMRP3116	*Cladosporium* sp.	MT075666
				CMRP3114	*Ochroconis* sp.	MT075667
				CMRP3113	*Cladosporium* sp.	MT075668
				CMRP3001	Pyriculariaceae	MT080300
				CMRP3074	Chaetothyriales	MT080301
				CMRP2985	Mycosphaerellaceae	MT080302
57	Decomposing Material	FOM	6	CMRP2986	Mycosphaerellaceae	MT080303
				CMRP2998	*Fonsecaea brasiliensis*	MT075669
				CMRP3104	Mycosphaerellaceae	MT080304
				CMRP3085	Mycosphaerellaceae	MT080305
				CMRP3107	Mycosphaerellaceae	MT080306
				CMRP3088	Chaetothyriales	MT080307
59	Decomposing Material	FOM	8	CMRP2855	Chaetothyriales	MT080308
				CMRP2856	Chaetothyriales	MT080309
				CMRP2865	Chaetothyriales	MT080310
				CMRP2869	Chaetothyriales	MT080311
				CMRP2874	Chaetothyriales	MT080312
				CMRP3002	Chaetothyriales	MT080313
				CMRP3109	*Fonsecaea brasiliensis*	MT075670
				CMRP2582	*Cladosporium* sp.	MT075671
71	Soil	FOM	1	CMRP2561	*Exophiala spinifera*	MT075672
72	Soil	FOM	5	CMRP2580	Mycosphaerellaceae	MT080314
				CMRP2602	*Exophiala spinifera*	MT075673
				CMRP2605	*Exophiala spinifera*	MT075674
				CMRP2610	Trichomeriaceae	MT080315
				CMRP3117	*Cyphellophora oxyspora*	MT075675
Total			67			

N, number of isolates; ID, identification; FOP, *F. pedrosoi* padlock probe; FOM, *F. monophora* padlock probe; CMRP, Coleções Microbiológicas da Rede Paranaense.

## Supplementary Materials

The following are available online at https://www.mdpi.com/2309-608X/6/4/290/s1, Table S1: Environmental samples from Maranhão state, Brazil, analyzed by RCA padlock probes.
