# Supplementary material for: Environmental Screening of Fonsecaea Agents of Chromoblastomycosis Using Rolling Circle Amplification
Source: J Fungi (Basel). 2020 Nov 17;6(4):290. doi: 10.3390/jof6040290 (PMC7712894; doi:10.3390/jof6040290)
Supplement: Supplementary file 1 [file jof-06-00290-s001.pdf]

SUPLEMENTARY TABLE

Environmental samples from Maranhão state, Brazil, analyzed by RCA padlock probes.

Sample	Padlock Probe		Source	Geography
	FOP	FOM		
1	+	+	Decomposing material	Bacabeira, Maranhão State, Brazil
2	+	+	Decomposing material	Bacabeira, Maranhão State, Brazil
3	-	-	Decomposing material	Bacabeira, Maranhão State, Brazil
4	-	+	Decomposing material	Bacabeira, Maranhão State, Brazil
5	-	-	Decomposing material	Bacabeira, Maranhão State, Brazil
6	-	+	Leaf of *A. vulgare*	Bacabeira, Maranhão State, Brazil
7	+	+	Leaf of *S.paniculatum*	Bacabeira, Maranhão State, Brazil
8	-	+	Leaf of *S. dulcis*	Bacabeira, Maranhão State, Brazil
9	-	+	Leaf of *M.paniculata*	Bacabeira, Maranhão State, Brazil
10	+	-	Leaf of *P. insignis*	Bacabeira, Maranhão State, Brazil
11	-	-	Plant spine of *A.vulgare*	Bacabeira, Maranhão State, Brazil
12	-	-	Plant spine of *S.paniculatum*	Bacabeira, Maranhão State, Brazil
13	-	-	Plant spine *S. paniculatum*	Bacabeira, Maranhão State, Brazil
14	-	-	Plant spine of *A. vulgare*	Bacabeira, Maranhão State, Brazil
15	-	-	Plant spine of *A. vulgare*	Bacabeira, Maranhão State, Brazil
16	-	-	Steam of, *A. vulgare*	Bacabeira, Maranhão State, Brazil
17	-	+	Steam of, *S.paniculatum*	Bacabeira, Maranhão State, Brazil
18	-	+	Steam of *S. dulcis*	Bacabeira, Maranhão State, Brazil
19	-	+	Steam of *M. paniculata*	Bacabeira, Maranhão State, Brazil
20	-	-	Steam of *P. insignis*	Bacabeira, Maranhão State, Brazil
21	-	-	Decomposing Material	São Benedito do Rio Preto, Maranhão State, Brazil
22	-	-	Decomposing Material	São Benedito do Rio Preto, Maranhão State, Brazil
23	-	-	Decomposing Material	São Benedito do Rio Preto, Maranhão State, Brazil
24	-	-	Decomposing Material	São Benedito do Rio Preto, Maranhão State, Brazil
25	-	+	Decomposing Material	São Benedito do Rio Preto, Maranhão State, Brazil
26	-	-	Leaf of *S.dulcis*	São Benedito do Rio Preto, Maranhão State, Brazil
27	-	-	Leaf of *M.paniculata*	São Benedito do Rio Preto, Maranhão State, Brazil
28	-	-	Leaf of *A. vulgare*	São Benedito do Rio Preto, Maranhão State, Brazil
29	-	+	Leaf of *A. vulgare*	São Benedito do Rio Preto, Maranhão State, Brazil
30	-	+	Leaf of *A.vulgare*	São Benedito do Rio Preto, Maranhão State, Brazil
31	-	-	Plant spine of *A. vulgare*	São Benedito do Rio Preto, Maranhão State, Brazil
32	-	-	Plant spine of, *A. vulgare*	São Benedito do Rio Preto, Maranhão State, Brazil
33	-	+	Steam of, *S. dulcis*	São Benedito do Rio Preto, Maranhão State, Brazil
34	-	+	Steam of *M. paniculata*	São Benedito do Rio Preto, Maranhão State, Brazil
35	-	+	Steam of *A.vulgare*	São Benedito do Rio Preto, Maranhão State, Brazil
36	-	+	Steam of *A. vulgare*	São Benedito do Rio Preto, Maranhão State, Brazil
37	-	+	Steam of *A. vulgare*	São Benedito do Rio Preto, Maranhão State, Brazil
38	-	-	Steam of *A.vulgare*	Nina Rodrigues, Maranhão State, Brazil
39	-	-	Steam of *S. dulcis*	Nina Rodrigues, Maranhão State, Brazil
40	-	-	Steam of *S. dulcis*	Nina Rodrigues, Maranhão State, Brazil
41	-	-	Steam of *S. dulcis*	Nina Rodrigues, Maranhão State, Brazil
42	-	-	Steam of *S. dulcis*	Nina Rodrigues, Maranhão State, Brazil
43	-	-	Leaf of *A. vulgare*	Nina Rodrigues, Maranhão State, Brazil
44	-	-	Leaf of *S. dulcis*	Nina Rodrigues, Maranhão State, Brazil
45	-	-	Leaf of *S. dulcis*	Nina Rodrigues, Maranhão State, Brazil
46	-	-	Leaf of *S. dulcis*	Nina Rodrigues, Maranhão State, Brazil
47	-	+	Leaf of *S. dulcis*	Nina Rodrigues, Maranhão State, Brazil
48	-	+	Steam of *A. vulgare*	Pinheiro, Maranhão State, Brazil
49	-	-	Steam of *Urtica* spp.	Pinheiro, Maranhão State, Brazil
50	-	-	Steam of *M. paniculata*	Pinheiro, Maranhão State, Brazil
51	-	+	Steam of *S. dulcis*	Pinheiro, Maranhão State, Brazil
52	-	-	Steam of *M. indica*	Pinheiro, Maranhão State, Brazil

53	-	-	Decomposing material	Nina Rodrigues, Maranhão State, Brazil
54	-	-	Decomposing material	Nina Rodrigues, Maranhão State, Brazil
55	-	-	Decomposing material	Nina Rodrigues, Maranhão State, Brazil
56	-	-	Decomposing material	Nina Rodrigues, Maranhão State, Brazil
57	-	+	Decomposing material	Nina Rodrigues, Maranhão State, Brazil
58	-	-	Decomposing material	Pinheiro, Maranhão State, Brazil
59	-	+	Decomposing material	Pinheiro, Maranhão State, Brazil
60	-	-	Decomposing material	Pinheiro, Maranhão State, Brazil
61	-	-	Decomposing material	Pinheiro, Maranhão State, Brazil
62	-	+	Decomposing material	Pinheiro, Maranhão State, Brazil
63	-	+	Leaf of *A. vulgare*	Pinheiro, Maranhão State, Brazil
64	-	-	Leaf of *Urtica* spp.	Pinheiro, Maranhão State, Brazil
65	-	-	Leaf of *M. paniculata*	Pinheiro, Maranhão State, Brazil
66	-	-	Leaf of *S. dulcis*	Pinheiro, Maranhão State, Brazil
67	-	-	Leaf of *M. indica*	Pinheiro, Maranhão State, Brazil
68	-	-	Soil	Bacabeira, Maranhão State, Brazil
69	-	-	Soil	Bacabeira, Maranhão State, Brazil
70	-	-	Soil	Bacabeira, Maranhão State, Brazil
71	-	+	Soil	Bacabeira, Maranhão State, Brazil
72	-	+	Soil	Bacabeira, Maranhão State, Brazil
73	-	-	Soil	São Benedito do Rio Preto, Maranhão State, Brazil
74	-	-	Soil	São Benedito do Rio Preto, Maranhão State, Brazil
75	-	+	Soil	São Benedito do Rio Preto, Maranhão State, Brazil
76	-	-	Soil	São Benedito do Rio Preto, Maranhão State, Brazil
77	-	+	Soil	São Benedito do Rio Preto, Maranhão State, Brazil
78	+	-	Soil	Pinheiro, Maranhão State, Brazil
79	-	-	Soil	Pinheiro, Maranhão State, Brazil
80	-	-	Soil	Pinheiro, Maranhão State, Brazil
81	-	-	Soil	Pinheiro, Maranhão State, Brazil
82	-	-	Soil	Pinheiro, Maranhão State, Brazil
83	-	-	Soil	Nina Rodrigues, Maranhão State, Brazil
84	-	-	Soil	Nina Rodrigues, Maranhão State, Brazil
85	-	-	Soil	Nina Rodrigues, Maranhão State, Brazil
86	-	-	Soil	Nina Rodrigues, Maranhão State, Brazil
87	-	-	Soil	Nina Rodrigues, Maranhão State, Brazil
88	+	+	Babassu coconut, *O. phalerata*	Maranhão State, Brazil [2]
89	-	+	Babassu coconut, *O. phalerata*	Maranhão State, Brazil [2]
90	-	+	Babassu coconut, *O. phalerata*	Maranhão State, Brazil [2]
91	-	+	Babassu coconut, *O. phalerata*	Maranhão State, Brazil [2]
92	-	+	Babassu coconut, *O. phalerata*	Maranhão State, Brazil [2]
93	-	+	Babassu coconut, *O. phalerata*)	Maranhão State, Brazil [2]
94	-	+	Babassu coconut, *O. phalerata*	Maranhão State, Brazil [2]
95	-	+	Babassu coconut, *O. phalerata*	Maranhão State, Brazil [2]
96	-	+	Babassu coconut, *O. phalerata*	Maranhão State, Brazil [2]
97	-	+	Babassu coconut, *O. phalerata*	Maranhão State, Brazil [2]
98	-	+	Babassu coconut, *O. phalerata*	Maranhão State, Brazil [2]
99	-	+	Babassu coconut, *O. phalerata*	Maranhão State, Brazil [2]
100	-	-	Babassu coconut, *O. phalerata*	Maranhão State, Brazil [2]
101	-	-	Babassu coconut, *O. phalerata*	Maranhão State, Brazil [2]
102	-	-	Babassu coconut, *O. phalerata*	Maranhão State, Brazil [2]
103	-	-	Babassu coconut, *O. phalerata*	Maranhão State, Brazil [2]
104	+	-	Babassu coconut, *O. phalerata*	Maranhão State, Brazil [2]
105	-	-	Babassu coconut, *O. phalerata*	Maranhão State, Brazil [2]
106	-	-	Babassu coconut, *O. phalerata*	Maranhão State, Brazil [2]
107	-	-	Babassu coconut, *O. phalerata*	Maranhão State, Brazil [2]

FOP- *F. pedrosoi* padlock probe; FOM- *F. monophora* padlock probe; (-) negative sample; (+) positive sample
